# Influence of zinc on the calcium carbonate biomineralization of *Halomonas halophila*

**DOI:** 10.1186/2046-9063-8-31

**Published:** 2012-12-01

**Authors:** Dirk Rothenstein, Johannes Baier, Thomas D Schreiber, Vera Barucha, Joachim Bill

**Affiliations:** 1 Institute for Materials Science, Heisenbergstraße 3, 70569, Stuttgart, Germany

## Abstract

**Background:**

The salt tolerance of halophilic bacteria make them promising candidates for technical applications, like isolation of salt tolerant enzymes or remediation of contaminated saline soils and waters. Furthermore, some halophilic bacteria synthesize inorganic solids resulting in organic–inorganic hybrids. This process is known as biomineralization, which is induced and/or controlled by the organism. The adaption of the soft and eco-friendly reaction conditions of this formation process to technical syntheses of inorganic nano materials is desirable. In addition, environmental contaminations can be entrapped in biomineralization products which facilitate the subsequent removal from waste waters. The moderately halophilic bacteria *Halomonas halophila* mineralize calcium carbonate in the calcite polymorph. The biomineralization process was investigated in the presence of zinc ions as a toxic model contaminant. In particular, the time course of the mineralization process and the influence of zinc on the mineralized inorganic materials have been focused in this study.

**Results:**

*H. halophila* can adapt to zinc contaminated medium, maintaining the ability for biomineralization of calcium carbonate. Adapted cultures show only a low influence of zinc on the growth rate. In the time course of cultivation, zinc ions accumulated on the bacterial surface while the medium depleted in the zinc contamination. Intracellular zinc concentrations were below the detection limit, suggesting that zinc was mainly bound extracellular. Zinc ions influence the biomineralization process. In the presence of zinc, the polymorphs monohydrocalcite and vaterite were mineralized, instead of calcite which is synthesized in zinc-free medium.

**Conclusions:**

We have demonstrated that the bacterial mineralization process can be influenced by zinc ions resulting in the modification of the synthesized calcium carbonate polymorph. In addition, the shape of the mineralized inorganic material is chancing through the presence of zinc ions. Furthermore, the moderately halophilic bacterium *H. halophila* can be applied for the decontamination of zinc from aqueous solutions.

## Background

A broad diversity of microorganisms affect and control geochemical processes like the mineralization of inorganic materials, which is known as biomineralization [reviewed in [1]. Such biominerals are hybrids of inorganic and organic components generating bones, teeth, or shells. The organic matrix, which consists of proteins, lipids, or polysaccharides, functions as template and/or nucleation site for the mineralization of the inorganic phase. Minerals which are synthesized by biomineralization processes include silica, iron oxides, hydroxyapatite, and calcium carbonate in various polymorph orientations, e.g. calcite, aragonite, and vaterite [2]. Calcium carbonate mineralization by bacteria is regarded as a biologically induced and mediated process [3]. The role of calcium carbonate biomineralization by bacteria is ambiguous. It is under debate if this mineralization process is an unwanted side effect of the metabolism under certain environmental conditions [4] or an intentional effect which is associated with environmental benefits for the microorganism [5]. Key factors which control the mineralization are the calcium concentration, the concentration of dissolved inorganic carbon (DIC), the pH of the surrounding solution, and available nucleation sites [6]. Microorganisms can influence most of the precipitation factors for the induction of the biomineralization process. Bacteria cells have been reported to act as nucleation sites or sites for calcium accumulation [7]. Positively charged ions, like Ca^2+^, can be accumulated on negatively charged functional groups on the cell surface and subsequently react with anions to form insoluble inorganic solids like calcium carbonate [5]. Metabolic pathways of heterotrophic bacteria, namely the nitrogen and the sulphur cycle, can be involved in biomineralization processes by the generation of (hydrogen-) carbonate ions and ammonia affecting the surrounding medium [8]. The synthesis of NH_4_^+^ leads to an increase of the pH of the environment which results in the shift of the carbonate-hydrogencarbonate equilibrium towards carbonate ions, which react with Ca^2+^ to form calcium carbonate.

Different genera of moderately halophilic bacteria were reported to mineralize calcium carbonate in natural habitats including the genus *Halomonas*. Moderately halophilic bacteria are a heterogeneous group of Gram-positive and Gram-negative aerobic as well as anaerobic bacteria [9]. They were found in various saline aquatic and terrestrial habitats, such as salterns, hypersaline soils, and lakes. Moderately halophilic bacteria grow under a wide range of salt concentrations and were also found in freshwater habitats [9]. The moderate halophilic bacterium *Halomonas halophila*, synonym *Deleya halophila*[10], is a member of the gram-negative *Halomonadaceae* family. The rod shaped bacteria have a salinity range between 2 and 30% sodium chloride with its optimum at 7.5%. *H. halophila* is aerobic and motile due to 1 to 8 flagella [10,11].

Halophilic microbes have evolved different strategies to overcome osmotic stress induced by high salt concentrations in the environment. Two basic mechanisms for osmoadaptation have been described: (1) the KCl type, which maintains a cytoplasmic KCl concentration similar to the given environmental conditions, and (2) the compatible solute type, using organic osmolytes also called compatible solutes [12]. These osmolytes are low-molecular weight organic compounds which balance the osmotic pressure and maintain a high intracellular turgor.

The biotechnological potential of moderately halophilic bacteria was explored for e.g. industrial applications of salt tolerant enzymes or the recovery of saline soil [9]. Biomineralization processes for the formation of organic–inorganic hybrid materials which have technical applications e. g. as nano-materials is currently in the focus of research. Thereby, the soft and ecofriendly reaction conditions of biomineralization shall be exploited for material generation. Furthermore, the regeneration of contaminated water and soil is still a pressing problem. Various industrial sectors, like tannery, chemical manufacturing, and petrochemical industry produce wastewaters containing high salt concentrations and metal pollutants. This presents a problem since conventional physico-chemical water remediation is cost intensive and biological treatments are not highly efficient yet [13]. Halophilic bacteria which biomineralize CaCO_3_ can accumulate toxic metal ions on the surface and may finally deplete such pollutants form the nutrient cycle by incrustation synthesizing CaCO_3_.

In order to investigate the influence of metal ions on the biomineralization process of moderately halophilic bacteria, *H. halophila* was adapted to high zinc concentrations in the environment. The biomineralization of calcium carbonate, in particular the initial stages of crystal growth, was monitored time resolved in the presence and absence of zinc ions.

## Results

### Influence of zinc ions on bacterial growth

**Figure 1:**
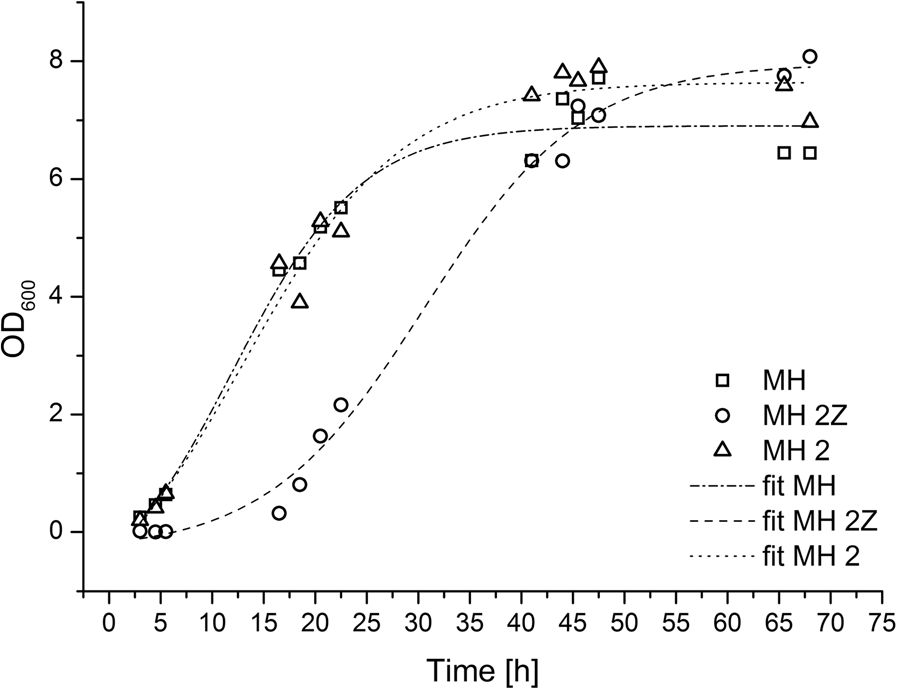
**Bacterial growth in the presence and absence of Zn ions.** The growth rate of bacteria in MH medium without calcium acetate (MH) and with Ca-acetate (MH2) was similar. After 3 hours the bacteria completed the lag phase and already entered the exponential growth phase, while in MH medium containing 0.3 mM Zn-acetate (MH 2Z) the lag phase was prolonged to approximately 7 hours. The growth of bacteria was not constrained afterwards. The growth was monitored by measuring the absorbance at 600 nm. Reading points were fitted with a sigmoidal function.

*Halomonas halophila* was adapted to zinc ions by increasing the concentration of zinc acetate in the culture medium to a final concentration of 0.3 mM. The investigations were performed in media with two different concentrations of calcium and in the presence or absence of zinc (Table 1). The zinc-adapted cultures showed a slightly prolonged lag phase of approximately 7 hours in medium with Zn^2+^ compared to a lag phase of > 3 hours of bacteria in medium without zinc supplementation (Figure 1). In cultures incubated longer than 45 hours the cell density was similar in media with and without zinc acetate. All investigated media alkalinized due to the metabolic activity of bacteria, the starting pH of 6.8 increased and reached a final pH value of approximately 8.7 after 7 days of cultivation.

**Table 1:** **Supplementation of basal medium**^**1**^

**Medium**	**Supplementation of the MH basal medium**^1^
MH 2	0.2% (w/v) Ca-acetate
MH 2Z	0.2% (w/v) Ca-acetate, 0.3 mM Zn-acetate
MH 4	0.4% (w/v) Ca-acetate
MH 4Z	0.4% (w/v) Ca-acetate, 0.3 mM Zn-acetate

^1^MH Basal medium (10 g/L yeast extract, 5 g/L peptone, 1 g/L glucose, 75 g/L NaCl).

### Concentration of zinc and calcium

The calcium and zinc ion concentrations were determined by inductively coupled plasma optical emission spectrometry (ICP-OES) over a culture period of 11 days, with measuring points at 1, 3, 7, 9, and 11 days. For each measurement the concentrations in the medium, for membrane associated zinc and calcium, and after lysis of the bacteria cells (soluble intracellular fraction) were determined. The concentrations of Zn^2+^ and Ca^2+^ after one day were determined for the medium only, because the amount of bacteria cells were insufficient to perform ICP-OES.

For all media the concentrations of zinc and calcium were determined before the experiment. The measured and the calculated values of the zinc (calculated: 20 μg/mL vs. measured: 19.41 μg/mL) and calcium (calculated: < 1000 and < 500 μg/mL vs. measured: 902.87 and 486.86 μg/mL) fit well. The concentrations of zinc and calcium in the buffers used for sample preparation (PBS, lysozyme /PBS, and HCl) were determined below the detection limit for both, zinc (< 0.05 μg/mL) and calcium (< 0.5 μg/mL), respectively. Therefore, the possibility of contaminating the samples with zinc and calcium due to the buffer formulations can be excluded.

**Figure 2:**
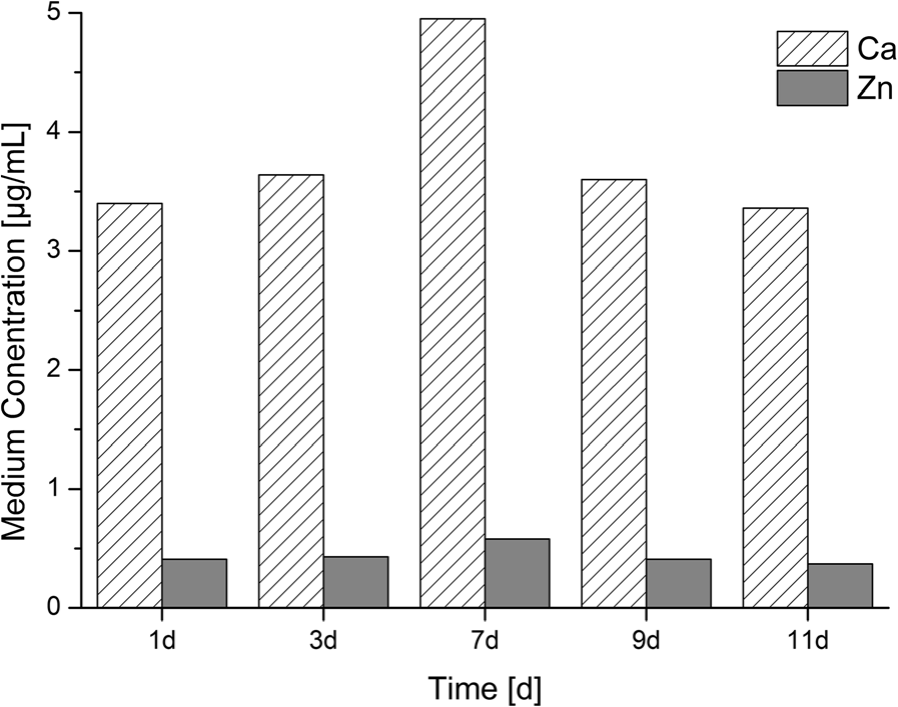
**Changes with time of zinc and calcium concentration in MH medium.** The concentrations of zinc and calcium in the medium did not show fluctuation and the mean concentrations showed only small standard deviations for calcium 3.98 ± 0.6 μg/mL and for zinc 0.44 ± 0.08 μg/mL.

For bacteria cultures in MH medium, which was supplemented neither with calcium nor zinc, the basal calcium and zinc concentrations were constant over the entire experimental period (Figure 2). The Ca^2+^ concentration was determined to be 3.98 ± 0.6 μg/mL and the concentration for zinc was 0.44 ± 0.08 μg/mL. Due to the low calcium concentration in the medium, the amount of Ca^2+^ associated with bacteria cells was marginal with a concentration of only 0.82 ± 0.17 μg/mL (data not shown).

**Figure 3:**
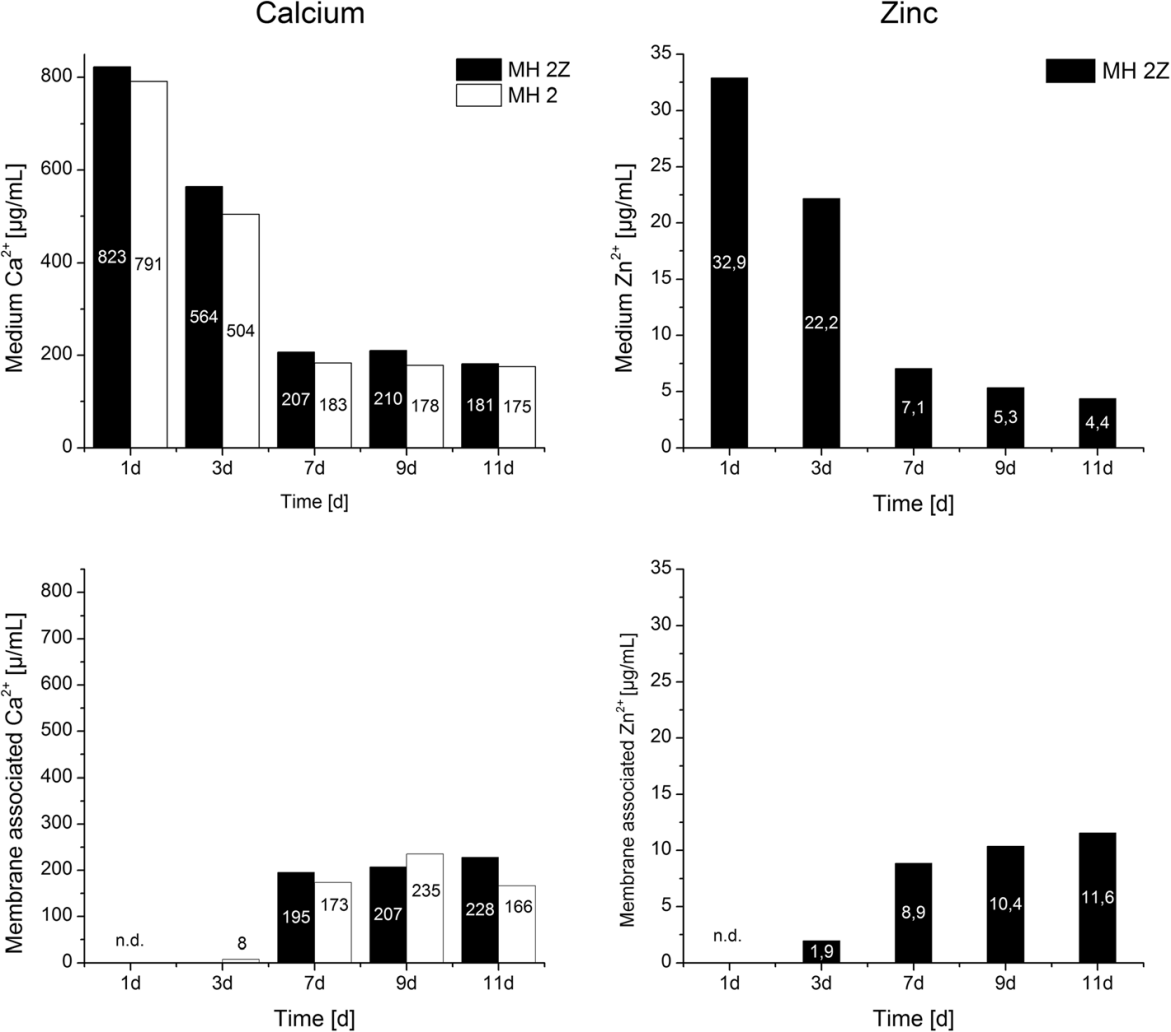
**Calcium and zinc concentrations were determined in media and membrane associated by ICP-OES. ***Halomonas halophila* were cultivated in MH 2 and MH 2Z medium. Samples at 1, 3, 7, 9, and 11 days were analyzed. Black columns: MH 2Z medium, white columns: MH 2 medium. Concentrations of calcium and zinc are indicated in the columns in μg/mL. The concentration of membrane associated calcium at day 1 was not determined (n.d.) due to cell density limitations.

**Figure 4:**
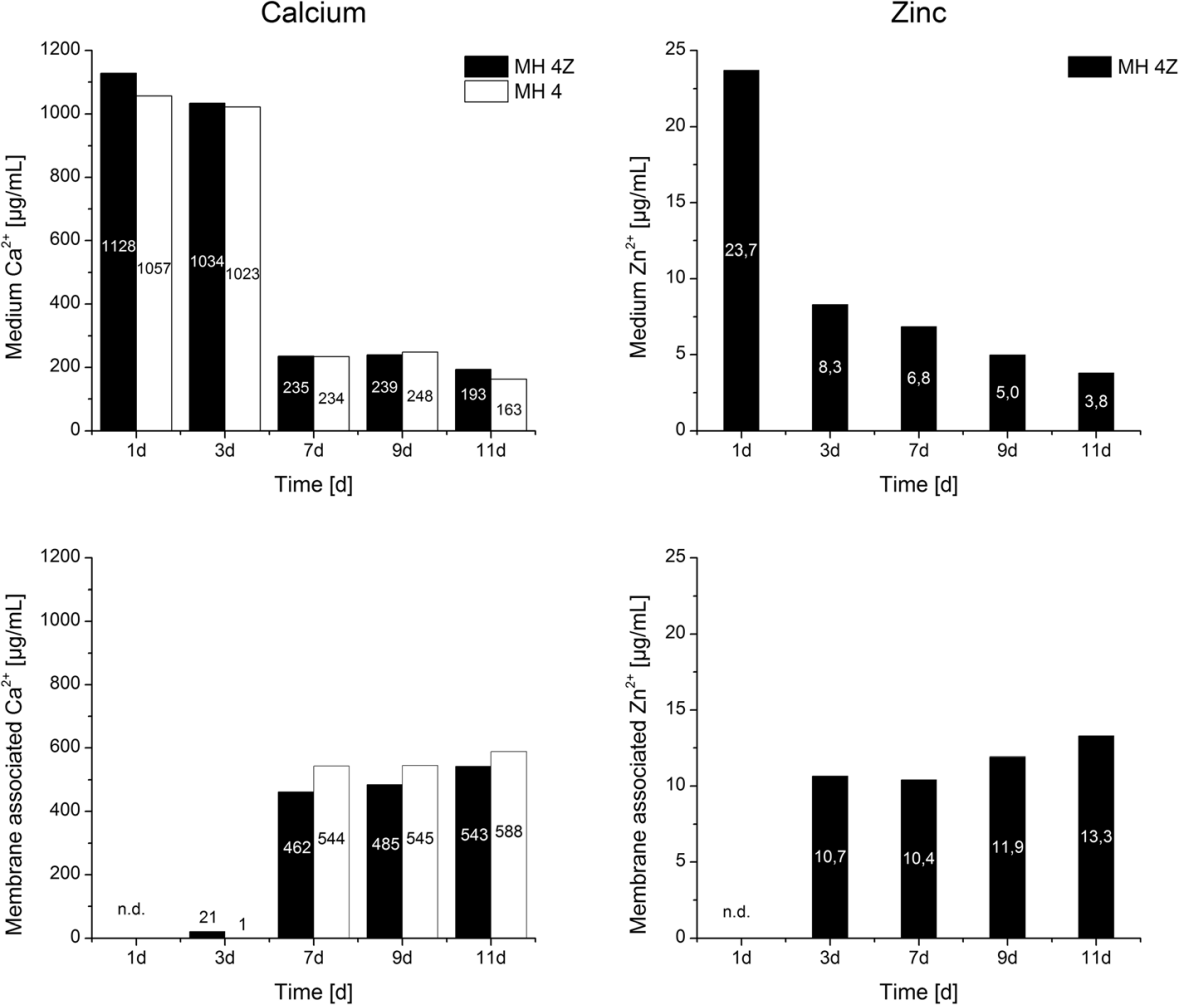
**Calcium and zinc concentrations were determined in media and membrane associated by ICP-OES. ***Halomonas halophila* were cultivated in MH 4 and MH 4Z medium. Samples at 1, 3, 7, 9, and 11 days were analyzed. Black columns: MH 4Z medium, white columns: MH 4 medium. Concentrations of calcium and zinc are indicated in the columns in μg/mL. The concentration of membrane associated calcium at day 1 was not determined (n.d.) due to cell density limitations.

**Figure 5:**
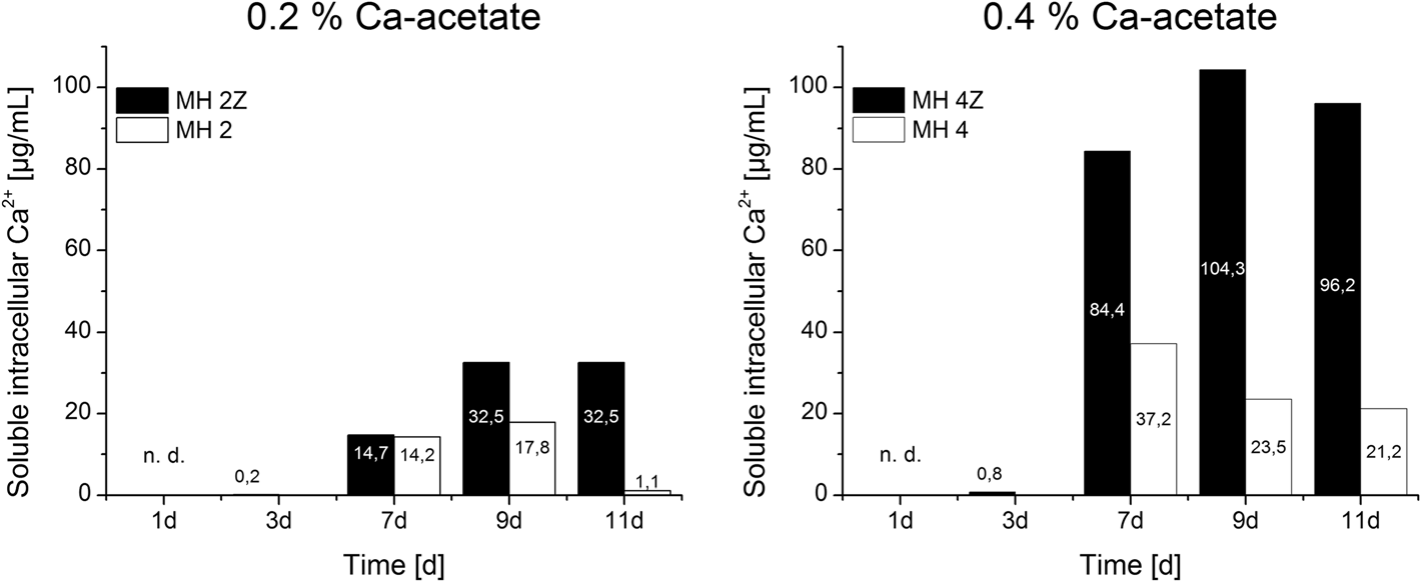
**Soluble intracellular calcium concentrations in media with 0.2% (left) and 0.4% (right) Ca-acetate concentrations.** The soluble intracellular calcium levels showed a dependency of the presence of zinc in the medium. Bacteria cells accumulated calcium to higher levels in medium with zinc acetate (MH 2Z and MH 4Z) compared to media without elevated zinc concentrations (MH 2 and MH 4). Concentrations of calcium are indicated in the columns in μg/mL. The concentration of calcium at day 1 was not determined (n.d.) due to cell density limitations.

The ion concentrations were determined for 5 mL of cultivated bacteria suspension. The densities of bacteria in the different cultivation media were similar after 45 h (Figure 1) which allows the comparison of the values. In media, which were supplemented with calcium acetate and/or zinc acetate the Zn^2+^ and Ca^2+^ ion concentrations changed with time during the bacterial growth and mineralization (Figures 3 and 4). The general trends in changes of Ca^2+^ and Zn^2+^ concentrations with time were similar in all four media. The calcium concentrations in the medium converge to a stable concentration of around 200 μg/mL from the seventh day following, regardless of the initial concentrations (Figures 3 and 4, top left). The main calcium depletion of the media was monitored in between the day 3 to day 7. The concentrations of calcium in medium MH 2 and MH 2Z at day 1 were elevated compared to the expected calculated values (Figure 3). The accumulation of membrane associated calcium depends on the initial Ca-acetate concentration in the medium. Higher Ca-acetate concentrations in the medium lead to higher Ca^2+^ accumulations. The membrane associated calcium concentrations showed a distinct calcium accumulation at day 7 and afterwards. While bacteria grown in MH 2 and MH 2Z media accumulated calcium to approximately 200 μg/mL at day 11 (Figure 3), bacteria in MH 4 and MH 4Z media showed values which were more than doubled and reach a value of 500 μg/mL (Figure 4). The concentrations of calcium of samples after cell lysis were considerably lower compared to the concentrations in the medium and for the surface immobilized calcium (Figure 5). The calcium ion concentrations after cell lysis also reflect the different media formulations. In media MH 2 the concentration of soluble intracellular Ca^2+^ was lower compared to MH 4 medium. Interestingly, in media with zinc supplementations the intracellular calcium concentrations were increased. The ICP-OES measurements of the soluble intracellular Ca^2+^ concentrations suggested that the Ca^2+^ levels in MH 2Z medium and in MH 4Z medium were elevated compared to the corresponding media without zinc (Figure 5).

The zinc concentrations in the media decreased till the end of the experimental period (Figures 3 and 4, top right). The final concentrations were determined of around 15% of the initial zinc amount for both media which is an 85% depletion of Zn^2+^ from the medium. The changes of Zn^2+^ concentrations with time in the medium with low (MH 2Z) and high (MH 4Z) calcium acetate concentrations did not significantly differ among each other. In both media similar amounts of zinc were adsorbed on the bacterial surface and simultaneously depleted from the medium. This process was active until the end of the experiment, showing a steady depletion of Zn^2+^ from the media and an enrichment in membrane associated Zn^2+^. Therefore, it can be concluded, that the Ca^2+^ concentration in the medium has a marginal effect on the depletion of Zn^2+^ from the medium and adsorption on the bacteria surface. The Zn^2+^ concentrations after lysis of bacteria cells were below the detection limit for both conditions (data not shown).

### SEM

Bacteria induced the formation of inorganic biominerals in MH medium supplemented with Ca-acetate and in media with additional Zn-acetate supplementation. The formation of biominerals was monitored over the first 21 days of cultivation. In media without *H. halomonas* no mineral phase was observed under the same conditions. Without Ca-acetate in the cultivation medium (MH medium) bacteria cells show small particles on the cell surface which might originate from the basal calcium content (4 mg/L) in the MH medium. Only in media with high Ca^2+^ concentrations (MH2, MH2Z, MH4, and MH4Z) the formation of a mineral phase was monitored.

**Figure 6:**
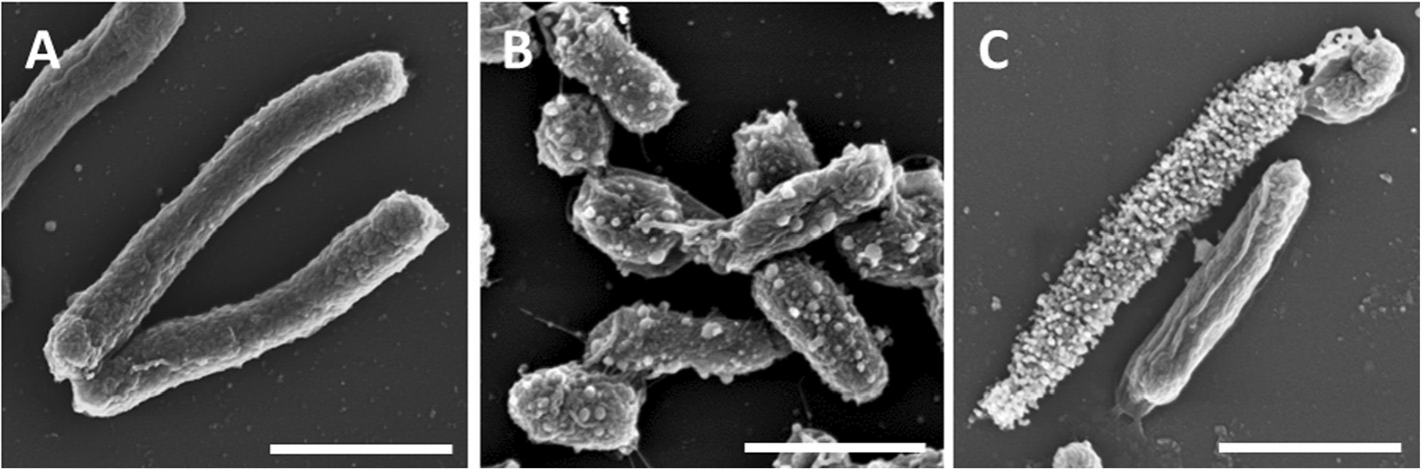
**Time resolved mineralization of calcium carbonate in medium with and without Zn**^**2+**^**.** The initial stages of the mineral formation show no differences between the cultures in media with and without Zn^2+^ and various Ca-acetate concentrations. **A**) After 24 h bacteria show no sign of accumulation of inorganic particles on the cell surface. **B**) and **C**) In the further course of the cultivation (7 days), bacteria accumulate globular inorganic particles in different amounts on the cell surface. Bars represent 1 μm.

The mineralization processes were similar for all investigated media within the first seven days of cultivation (Figure 6). In this period, no differences in the shapes of the mineralized inorganic materials were monitored. After 24 hours of cultivation no visible accumulations of inorganic material on the bacterial cell surfaces was determined (Figure 6A). The first inorganic particles associated with the bacteria were monitored after 7 days of cultivation. On bacteria cells in all media single to multiple small spherical particles, which possess a maximum diameter of approximately 70 nm were deposited (Figure 6B and 6C). The number of the accumulated particles varies from only a few to densely packed layers covering whole bacteria cells. All particles which were investigated show a uniform globular shape. The supplementation of the media with zinc acetate apparently did not affect the morphology of inorganic materials in early stages of biomineralization.

**Figure 7:**
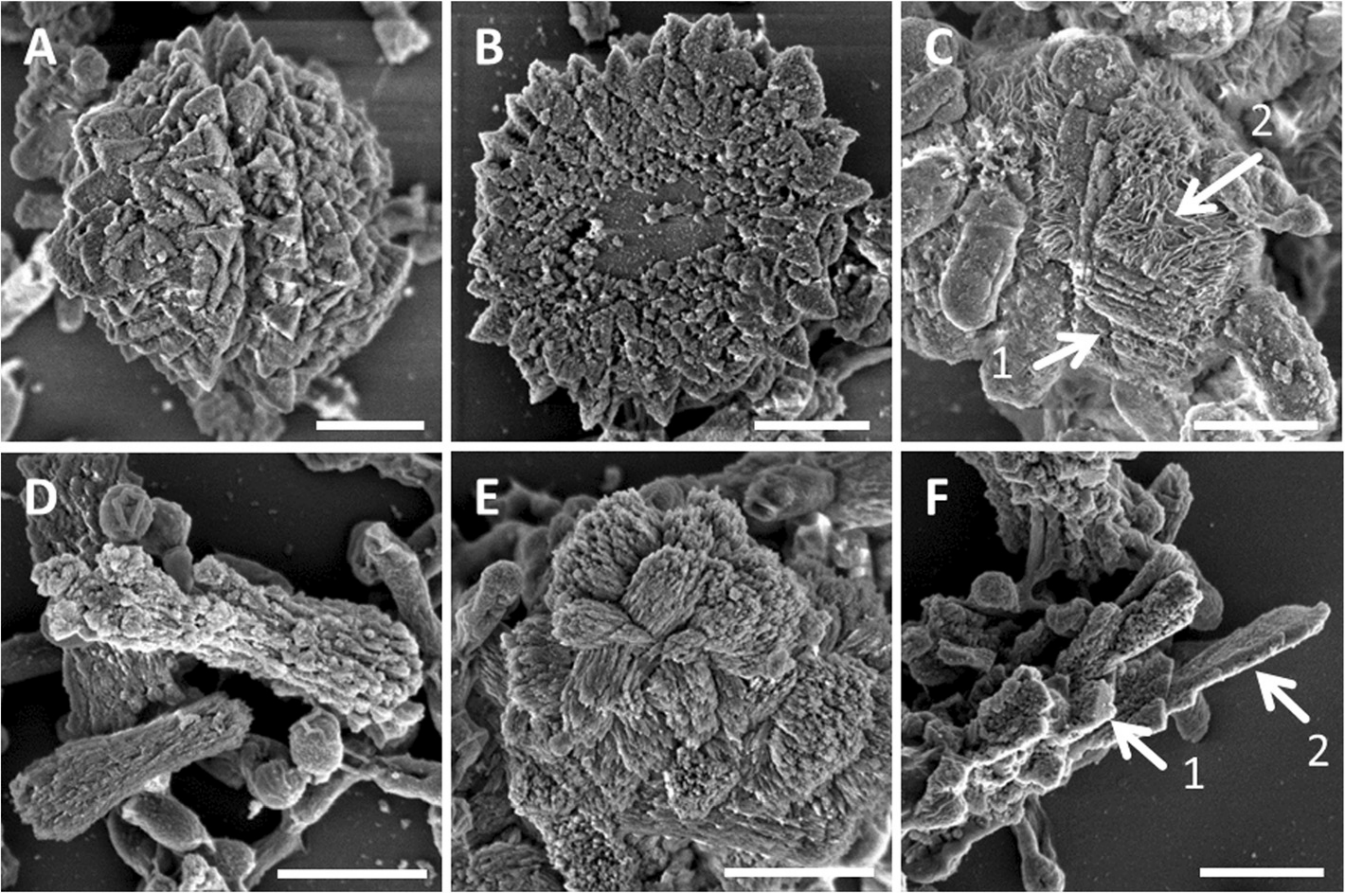
**Morphology of mineralization products.** Morphological variations of the mineralized CaCO_3_ after 20 days in medium with and without Zn^2+^. **A**) In MH 2 medium triangular CaCO_3_ platelets were mineralized and assemble into globular agglomerates with equatorial constrictions. **B**) Cross sections of these agglomerates show the internal construction, which consists of two clearly distinct regions. A smooth inner region is surrounded by an outer region which is composed of triangular platelets and globular particles. The inner region might have been the localization site of bacteria that have been the starting point for particle agglomeration. **C**) In MH 2Z medium (with Zn^2+^) particles show a mixed structure of platelet (arrow 1) and sponge like structures (arrow 2). **D**) In MH 4 medium the initial globular inorganic particles on the bacterial cells elongated and form columnar structures. **E**) In addition to densely packed needle-like structures. **F**) In MH 4Z medium only irregular agglomerates consisting of columnar structures (arrow 1) and sheet like structures (arrow 2) are mineralized. Bars represent 1 μm.

In the further scope of mineralization the inorganic particles show morphological differences (Figure 7). In MH 2 medium triangular plates were mineralized (Figure 7A and 7B). These platelets often form composed globular agglomerates which show an equatorial constriction (Figure 7A). Agglomerates split at the constriction, which is similar to a cross section, revealed an inner morphology of two distinct regions (Figure 7B). A smooth inner part and a rougher outer region assembled of triangular particles. The inner region might have been the localization site of bacteria cells which functioned as starting points for the agglomeration of inorganic CaCO_3_. Beside agglomerates of triangular platelets, rarely sponges like particles were observed. The particles shapes changed in MH 2Z medium (Figure 7C). Mixed particles consisting of stacked platelets (Figure 7C, arrow 1) and sponge like areas (Figure 7C, arrow 2) were mineralized. At higher Ca-acetate concentrations in the MH 4 medium the mineralization products show a distinct structure compared to all other media (Figure 7D and 7E). Columnar structures (Figure 7D) and bundles of needle like structures oriented in different spatial directions (Figure 7E) were monitored. In MH 4Z medium elongated and irregular shaped agglomerates were mineralized (Figure 7F). The composition of these agglomerates often showed that the sheet like structures intermingled irregular shaped areas.

### EDX analysis

**Figure 8:**
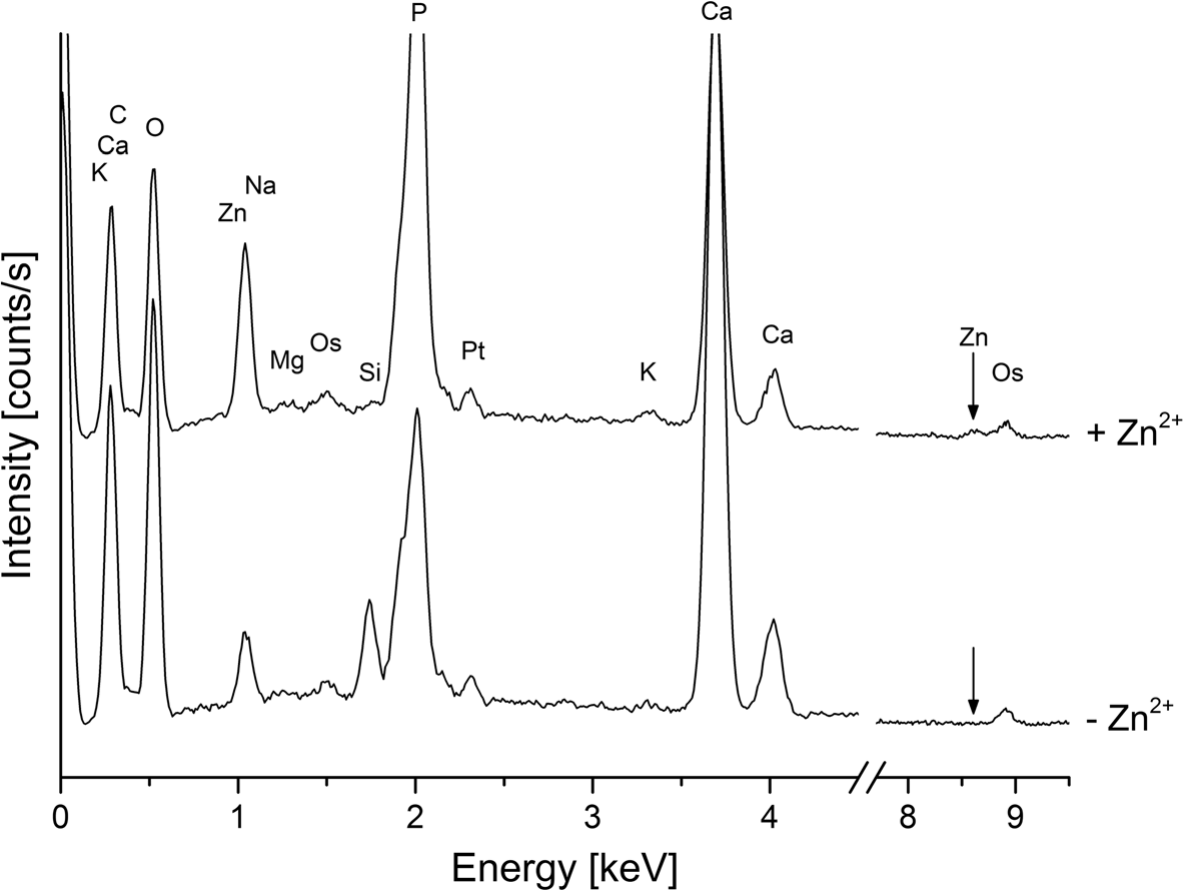
**Elemental analysis of bacteria cells and the mineralized inorganic phase by EDX spectroscopy.** For both culture media calcium accumulated to high levels, reflecting the formation of calcium carbonate. In medium with zinc acetate supplementation (upper spectrum, + Zn^2+^) additional zinc peaks were detected compared to the one without zinc (− Zn^2+^). The detection of osmium and platinum is due to the subject of sample preparation and staining. The silicon peak originates form the silicon wafer which was used as sample holder.

The elemental compositions of the mineralization products were determined by EDX analysis (Figure 8). The amounts of the inorganic material at the initial stages of biomineralization on single bacteria cells were generally too low for EDX analysis. Therefore, samples with dense bacteria layers were prepared to perform EDX analysis of organic and inorganic components. The presence of osmium and palladium were on account of the sample preparation while the silicium signal originates from the silicon wafer which was used as sample support.

EDX signals for calcium were absent in medium without Ca-acetate (data not shown). In medium with 0.2% Ca-acetate (MH 2 and MH 2Z) calcium accumulated to high levels which reflected the formation of biominerals. Furthermore, in MH 2Z medium zinc was additionally detected, suggesting the association of zinc with the bacteria cells.

### XRD

**Figure 9:**
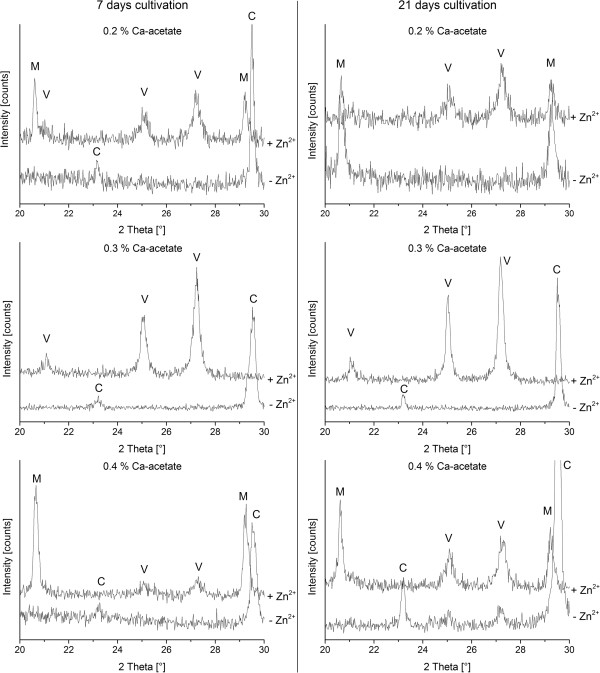
**XRD measurements of mineralized inorganic phases in media without (−Zn**^**2+**^**) and with (+Zn**^**2+**^**) zinc acetate supplementation.** Bacteria in media without Zn-acetate supplementation and at all Ca-acetate concentrations mineralize only calcite after 7 days of cultivation. Samples mineralized 21 days have various calcium carbonate phases. In medium with 0.3% Ca-acetate calcite (C) is the only mineralized crystal form while for 0.4% Ca-acetate a small amount of vaterite (V) was detected in addition. In the medium containing 0.2% Ca-acetate monohydrocalcite (M) was precipitated. Media with Zn-acetate supplementation and 0.2% as well as 0.4% Ca-acetate mineralized monohydrocalcite and vaterite after 7 and 21 days of cultivation. In samples from 0.3% Ca-acetate only vaterite was formed.

The polymorph of the mineralized inorganic phase was determined by X-ray diffraction (Figure 9). In order to deepen the understanding of the concentration dependence of the inorganic phase formation additional media with 0.3% Ca-acetate with (MH 3) and without zinc acetate supplementation (MH 3Z) were included in the experiments. All samples were analyzed after 7 and 22 days of cultivation (Table 2). In the majority of cases, the mineral polymorph did not change with time. Only in medium MH 2 we observed the transition from calcite at day 7 to monohydrocalcite at day 22. In all other media containing only calcium acetate (MH 3 and MH 4), calcite was the only polymorph which was mineralized at 7 and 22 days of cultivation. In addition, samples from MH 4 medium at 22 days of cultivation showed traces of vaterite. In media with zinc acetate different polymorphs of calcium carbonate were biomineralized. Monohydrocalcite and vaterite were synthesized and mixtures of both polymorphs were also identified. Only in MH 3Z medium vaterite was the exclusively mineralized inorganic phase.

**Table 2:** Mineralized calcium carbonate polymorph

**Medium**	**7 days of cultivation**	**22 days of cultivation**
MH 2	Calcite	Monohydrocalcite
MH 2Z	Monohydrocalcite Vaterite	Monohydrocalcite Vaterite
MH 3	Calcite	Calcite
MH 3Z	Vaterite	Vaterite
MH 4	Calcite	Calcite
MH 4Z	Monohydrocalcite Vaterite	Monohydrocalcite Vaterite

## Discussion

The biogenic mineralization of calcium carbonate (CaCO_3_) by *H. halophila* in the presence of zinc was investigated. *H. halophila* maintained the ability of CaCO_3_ mineralization in a Zn^2+^ contaminated environment. The bacteria function as inducers for the mineralization of calcium carbonate mineralization since in media without bacteria no mineralization of CaCO_3_ was observed. The inorganic phase was mineralized in media with high calcium concentrations only. The basal Ca^2+^ concentration (around 4 mg/L) in the MH medium, which has no calcium supplementation, is not sufficient to mineralize calcium carbonate in high amounts. Bacteria that are cultured in MH medium show the formation of isolated particles on the cell surface. Spontaneous mineralization of calcium carbonate in medium in the absence of *H. halophila* was not observed in the experimental setup. *H. halophila* which was adapted to zinc in the medium still mineralizes calcium carbonate. In the presence of Zn^2+^ the formation of calcite was suppressed, which is mineralized in MH 2 and MH 4 media, instead vaterite and monohydrocalcite were synthesized (Table 2). Functional groups on the cell surface may lead to changes in nucleation energy and thus induce the mineralization process [14]. The onset of significant mineralization activity that is associated with the decrease of calcium from the medium was determined for cultivation times longer than 3 days. Within the first 6 days the major decrease of calcium from the medium was monitored. In parallel, the accumulation of membrane associated calcium increases significantly from day 7 onwards which is indicative for the mineralization of CaCO_3_. A similar time frame for the mineralization of calcium carbonate by Flavobacterium was reported [15]. Flavobacterium strains show the induction of calcium carbonate mineralization between 3 and 7 days of cultivation in medium with 7.5% salt concentration at 22°C. The mineralization process of Flavobacterium and *H. halophila* both result in the formation of calcite [16].

Beside the mineralizing organism, the mineralization process is highly dependent on environmental conditions, e.g. temperature, pH, ionic strength [6]. Various microorganisms show different optimal mineralization conditions. High salt concentrations negatively affect the biomineralization process of *H. halophila*[16] and other moderately halophilic bacteria strains (e.g. Flavobacteria, Acinetobacter) [15]. Interestingly, other moderately halophilic bacteria strains have an optimal external salt concentration of 10% to 20% for the biomineralization of calcium carbonate and the formation of inorganic crystals is suppressed under low salt concentrations [17]. This indicates that beside environmental conditions bacteria actively influence the mineralization process. Furthermore, Hammes and Vestraete [6] stated that microorganisms can influence most mineralization factors, e.g. pH, local calcium concentration by surface adsorption, concentration of dissolved inorganic carbon, and therefore have some control over the biomineralization process. One of the influencing parameters is the pH value in solution. The pH value in the media increased to alkalinity (around pH 9) during the cultivation of *H. halophila*. The bacterial metabolic process generates a global alkaline environment. In particular the metabolism of organic nitrogen, like the aerobic oxidative deamination of amino acids and the reduction of nitrate, leads to the increase of the pH value in the surrounding environment [18]. The pH shift of the medium from neutral to alkaline conditions facilitates the precipitation of calcium carbonate. Moreover, bacteria cells have been reported to act as nucleation sites or sites for calcium accumulation [7]. Positively charged ions (e.g. Ca^2+^) can be accumulated at negatively charged functional groups on the cell surface and subsequently react with anions to form insoluble materials like calcium carbonate [5]. The experimental set up showed that the amount of surface bound calcium depends on the initial Ca-acetate concentration in the medium. High calcium concentrations in the medium leads to a high membrane associated calcium concentration. Zn^2+^ in the medium does not influence the accumulation of Ca^2+^ on the bacteria cells which is in accordance to the continuity of the biomineralization process in the presence of zinc.

The soluble intracellular calcium concentrations were similar in medium with low and high Ca^2+^ concentrations. In contrast to the membrane-associated calcium concentrations, zinc apparently influences the intracellular calcium concentration. In both media containing Zn^2+^ (MH 2Z and MH 4Z) the concentrations of calcium was significantly increased in the cell lysate compared to the corresponding media without Zn^2+^. The levels of cytoplasmic free Ca^2+^ are strictly regulated in bacteria cells, since it is assumed to play a role in chemotaxis, cell division and signal transduction [19]. Ca^2+^ levels in *E. coli* cells range between 200 to 300 nM with 2 to 7 fold fluctuations on external calcium concentration changes [20]. Interestingly, bacteria cells in the stationary phase appear to have less control over internal free Ca^2+^[20]. The cytoplasmic Ca^2+^ levels can be regulated with transporter systems like the pH dependent Ca^2+^/H^+^ antiporter [21] or the inorganic phosphate co-transporter [22]. Also, a polyhydroxybutyrate-polyphosphate (PHB) complex in *E. coli* was reported that can accumulate large amounts of Ca^2+^ in addition to function as a specific Ca^2+^ channel [19]. Our results suggest that zinc ions affect Ca^2+^ homeostasis leading to high intracellular calcium concentrations (Figure 5). The effect might be based on interference of Zn^2+^ with calcium transporter systems which regulates the intracellular calcium levels.

Zinc was only detected in the medium or on the surface of bacteria cells. The levels of zinc in lysed bacteria cell samples were below the detection limit. The main fraction of the zinc ions in solutions was accumulated on the bacterial cell surface by biosorption and removed from the environment leading to the depletion of zinc in the medium. Contrasting this extracellular immobilization of zinc ions, microalgae incorporate zinc and decontaminate it by the formation of zinc-phosphate based nano needles [23]. Although zinc is required for many processes in living organisms high intracellular zinc concentrations are toxic. Therefore, various cellular systems have evolved to maintain zinc homeostasis in bacteria cells. In bacteria members of the HME-RND (heavy metal efflux - resistance, nodulation, cell division) protein family, CDF (cation diffusion facilitators) family, and P-type ATPases were identified which are involved the export of Zn^2+^[24]. Furthermore, the efficient cell surface binding of zinc ions might also contribute to low intracellular Zn^2+^ levels. Based on the bacterial sorption model of Fein *et al.* acidic (pKa < 4.7, e.g. carboxyl and phosphodiester), neutral (pKa ≈ 7, e.g. phosphomonoester) and basic sites (pKa > 8, e.g. hydroxyl and amine groups) are involved in metal binding [25]. The Zn^2+^ adsorption was reported primarily to carboxyl- and phosphate-type functional groups [26]. Zinc homeostasis mechanisms and cell surface binding of zinc may be responsible for maintaining intracellular zinc concentrations below the detection limit.

Calcite was the predominant polymorph which was mineralized in media in the absence of Zn^2+^. The formation of calcite in media containing NaCl as sole salt is in agreement with earlier reports [27,28]. The mineralized polymorph is not only dependent on environmental conditions (e.g. ionic strength, pH, temperature) but is also dependent on the biomineralizing bacteria strains [15]. Under the same experimental conditions Flavobacterium and Acinetobacter stains mineralized other CaCO_3_ polymorphs. Furthermore, the Ca-acetate concentration in the media showed no effect on the mineralized polymorph, calcite was predominantly mineralized. Surprisingly, in MH 2 medium monohydrocalcite, which is thermodynamically less stable than calcite, was detected at 22 days of cultivation. In precipitation experiments without organic material, monohydrocalcite precipitates at a solution saturation state which is significantly lower than the saturation state of solutions precipitating calcite [29]. Furthermore, the precipitation of calcite promoted the dissolution of monohydrocalcite, suggesting the transition of monohydrocalcite to calcite [29]. In summary, the mineralization of monohydrocalcite starts at low Ca^2+^ concentrations and precedes the formation of calcite which is mineralized after the accumulation of high amounts of Ca^2+^. Our biomineralization experiments did not show in general a periodic change of mineralization products. Rather monohydrocalcite is stabilized either by zinc ions or by organic–inorganic interfacial interactions. In medium with Zn^2+^ the mineralization of calcite was suppressed. The predominant polymorphs were vaterite and monohydrocalcite. Under non-biological conditions, vaterite transforms quickly into calcite, which is the more stable phase of calcium carbonate. Using the double diffusion technique for the synthesis of calcium carbonate in the absence of organic additives at pH values between 10.4 to 10.8, calcite was generated while in the presence of Zn^2+^ aragonite was precipitated [30]. The Zn^2+^ cations were assumed to inhibit the transformation of the aragonite to the stable polymorph calcite [30,31]. The biomineralization in the absence of zinc resulted in the mineralization of calcite, similar to the synthesis in the absence of bacteria, while in the presence of zinc monohydrocalcite and vaterite were generated. Since in double diffusion experiments aragonite and not monohydrocalcite or vaterite was precipitated, our results indicate that the bacteria additionally influence the mineralized polymorph. It was also reported, that natural deposits of vaterite are most often associated with biogenic activity [32]. Organic molecules might stabilize and/or favor the vaterite formation due to (1) organic templates that induce the heterogeneous vaterite mineralization [32] or (2) the action of organic molecules that inhibit the transformation of the metastable vaterite to stable phases [33]. The mineralization and stabilization of the less stable CaCO_3_ polymorphs in our experiments might be accounted on these phenomena, too. In *Bacillus licheniformis* S-86 cultures, the extracellular polymeric substance (EPS) induces the agglomeration of bacteria cells in solution [26], which was also monitored in the cultivation of *H. halophila* in our experiments. In mineralization experiments with *B. licheniformis* S-86 producing EPS as well as in EPS solutions without bacteria calcite was mineralized. It was proposed that dissolved organic carbon (DOC) released from the EPS complexes Ca^2+^ ions in solution which reduces high supersaturation states which favor the formation of vaterite to lower Ca^2+^ levels which enhance the precipitation of calcite [14].

The morphology of the mineralized inorganic particles is divers, exhibiting globular, sponge-like, and triangular shapes. Interestingly, no defined inorganic structure can be correlated to a distinct CaCO_3_ polymorph. This was also shown for biogenic mineralized calcite and aragonite polymorphs which were morphologically not discriminable by electron microscopy [15]. The generated calcite agglomerates in MH 2 medium, consisting of triangular platelets exhibit a smooth inner part (Figure 7B). This region might be attributed to localization sites of bacteria, which initiated the mineralization process and became embedded during the mineralization process. Similar defects in biomineralized calcium carbonate crystals were reported [14]. Calcium carbonate crystals were pitted by bacteria-shaped holes which were assumed to be formed as a consequence of the deposition of mineralization products on the cell surface.

The biomineralization of calcium carbonate and also other inorganic materials can be classified into two different processes: (I) biologically induced and (II) biologically controlled mineralization [32]. The two processes differ regarding the degree of biological and genetic control. The mechanism (II) is generally more strictly regulated. The microalgae *Scenedesmus obliquus* mediate extracellular calcite formation in a biologically induced mineralization process. In the presence of Zn^2+^ the calcite polymorph is suppressed and aragonite is synthesized [34]. Zinc ions affect the biomineralized CaCO_3_ polymorph in both, algae and halophilic bacteria. Organisms mineralizing CaCO_3_ under biological control are e.g. gastropods and sea urchin larvae. There the mineralization takes place in a confined compartment inside the cell and organism, respectively. The mineralization of nacre in gastropods, a highly structured assembly of aragonite platelets and organic components, is controlled by organic template structures and soluble proteins [reviewed in [35]. The template forms a compartment of equally spaced layers in which the aragonite is mineralized. The organic molecules strictly regulate the polymorph, morphology, and nucleation of the inorganic material. In sea urchin larvae, spicules (skeleton) are synthesized in primary mesenchyme cells (PMC) starting from the 16-cell stage [36]. In early stages the skeleton consists of amorphous calcium carbonate which is stabilized by proteins. In the further development the amorphous phase transforms into calcite [32]. In the presence of Zn^2+^ the spicule formation is suppressed [36]. Compared to the biologically induced mineralization process in halophilic bacteria and algae, zinc has a fatal effect in spicule formation. For cadmium, gold, and silver it was suggested, that biominerals play also a role in detoxification processes by immobilization of adverse ions [37]. Here, we showed that for zinc contaminations the biologically induced mineralization in halophilic bacteria have a similar effect.

The mineralization of inorganic materials by moderately halophilic bacteria can be specifically modulated in the presence of zinc ions. These investigations show that bacterial mineralization processes might be exploiting for applications, like the remediation of wastewater or the generation of functional materials for technical use.

## Conclusions

In this report the biomineralization of calcium carbonate by the moderately halophilic bacterium *H. halomonas* was investigated. The bacteria can be adapted to metal ions contaminations like Zn^2+^ in the cultivation medium without a devastating influence on the growth rate. The biomineralization process in medium without zinc is reflected by the depletion of Ca^2+^ from the medium and its accumulation on the cell surface, which was demonstrated for different initial calcium concentrations in the medium. In addition, in medium with zinc acetate, the zinc ion concentration in the medium was minimized in the medium upon biomineralization. In parallel, zinc ions accumulate on the bacterial surface. The initial stages of biomineralization monitored by SEM were similar in the absence and presence of zinc ions. In the further course of the mineralization process, various shapes of inorganic material evolved. XRD measurements clearly showed that the presence of zinc ions influences the polymorph of the mineralized calcium carbonate, resulting in monohydrocalcite and vaterite. Since in precipitation experiments zinc ions lead to the formation of aragonite, the different polymorph is not only an effect of zinc ions, but is also controlled by the bacteria.

This approach shows that the polymorph of biomineralized inorganic materials can be changed in the presence of metal ions. In this process the metal ions were trapped on the bacterial surface and thus removed from the medium. Therefore, *H. halophila* is a candidate strain for the decontamination of saline waste water. In addition the bacteria are applicable for the generation of nanostructured inorganic materials by the biomineralization process.

## Methods

### Microorganisms

*Halomonas halophila* (DSM No 4770, Type strain) synonym *Deleya halophila* (CCM 3662) [10] a moderately halophilic bacterium which was isolated from hyper saline soil was used in this study. *H. halophila* is an aerobic, gram-negative rod-shaped bacterium with peritrichous flagella.

### Culture media and cultivation

Liqid MH medium, according to Rivandeneyra *et al.*[27], composed of 1% (w/v) yeast extract, 0.5% (w/v) proteose peptone, 0.1% (w/v) glucose, and 7.5% (w/v) sodium chloride. The medium was autoclaved and afterwards supplemented with filter sterilized 0.2, 0.3 or 0.4% (w/v) calcium acetate and/or 0.3 mM zinc acetate dihydrate. See Table 1 for nomenclature.

Liquid media assay *H. halophila* was inoculated in 25 mL medium at 26°C and 100 rpm. The biomineralization of calcium carbonates in the cultures was investigated after one, three, seven, nine, eleven, and 20 days. Bacterial growth was monitored by measuring the absorbance at 600 nm.

### Differential sample preparation for ICP-OES analysis

Ca- and Zn-ion concentrations were determined using inductively coupled plasma optical emission spectrometry (ICP-OES). Samples were prepared after one, three, seven, nine, and eleven days of culturing in order to determine the ion concentrations in the medium and associated with bacteria cells. Samples representing ion contents of medium, extracellular, intracellular, and cell debris were prepared.

Bacteria cells were removed from 5 mL culture medium by centrifugation (10 min, 4000 rpm). The pelleted bacteria were washed with phosphate buffered saline (PBS) and centrifuged again. To prepare the “medium sample” the supernatants (culture medium and PBS) were pooled and the solvent was evaporated. 1 mL sulfuric acid was added and the sample was heated to 100°C. After cooling to room temperature 0.5 mL nitric acid was added and the sample was heated to 375°C. This step was repeated twice. Finally the sample was adjusted with ddH2O to a final volume of 25 mL.

The bacteria cells were washed with 25 mM HCl for 10 minutes to remove extracellular bound Ca- and Zn-ions. Bacteria cells were removed by centrifugation (extracellular sample).

Bacteria cells were resuspended in PBS and lysed by adding lysozyme to access the intracellular ion concentrations. The cell debris was removed by centrifugation. The solvent of the supernatant was evaporated and the remaining cell debris was dried at 37°C. Both samples were prepared for ICP-OES analysis as described above.

### Sample preparation for REM

Bacteria samples were immobilized on poly-L-lysine coated Si-wafers. Samples were washed with ddH_2_O to remove excessive salts from the medium. Finally, the bacteria cells were fixed in glutaraldehyde and osmium tetroxide and dehydrated in ethanol which was then removed by critical point drying. To enhance the contrast, samples were sputtered with gold or platinum/palladium. Samples were investigated using the Zeiss DSM 982 GEMINI scanning electron microscope (SEM) at 3 kV. Energy-dispersive X-ray spectroscopy (EDX) was performed at 20 kV.

### X-ray diffraction (XRD)

In order to remove organic components from mineralized bacteria suspensions, samples were incubated with lysozyme and SDS solution and washed twice in ddH2O. Purified precipitates were transferred to Si-wafer and dried at 37°C. The structures of mineralized crystals were analyzed by X-ray diffraction with a Siemens D500 diffractometer with a Cu K_α_ radiation. 2θ was measured between 2° and 60° in steps of 0.02°.

## Competing interests

The authors declare that they have no competing interests.

## Authors’ contributions

DR conceived the study, analyzed data, and wrote the manuscript. JB analyzed the XRD data and participated in the design of the study and to draft the manuscript. TDS established the work with *H. halophila* and adapted the strain to zinc. VB carried out the culture experiments and the sample preparation for SEM, ICP-OES, and XRD analysis. All authors have read and approved the final manuscript.
